# Formoterol reduces muscle wasting in mice undergoing doxorubicin chemotherapy

**DOI:** 10.3389/fonc.2023.1237709

**Published:** 2024-01-03

**Authors:** Edson Alves de Lima Junior, Alexandre Abilio de Souza Teixeira, Loreana Sanches Silveira, Queralt Jové, Natalia Álvarez Ladrón, Marcelo G. Pereira, Francisco Javier López-Soriano, Josep M. Argilés, Patrícia Chakur Brum, Silvia Busquets, José Cesar Rosa Neto

**Affiliations:** ^1^ Immunometabolism Research Group, Department of Cell and Developmental Biology, University of São Paulo, São Paulo, Brazil; ^2^ Cancer Research Group, Departament de Bioquímica i Molecular Biomedicine, Facultat de Biologia, Universitat de Barcelona, Barcelona, Spain and Institut de Biomedicina de la Barcelona (IBUB), Barcelona, Spain; ^3^ Leeds School of Biomedical Sciences, Faculty of Biological Sciences University of Leeds, Leeds, United Kingdom; ^4^ School of Physical Education and Sport, University of São Paulo, São Paulo, Brazil

**Keywords:** beta_2_-adrenergic agonist, doxorubicin, chemotherapy, Lewis lung carcinoma, muscle wasting

## Abstract

**Background:**

Even though doxorubicin (DOX) chemotherapy promotes intense muscle wasting, this drug is still widely used in clinical practice due to its remarkable efficiency in managing cancer. On the other hand, intense muscle loss during the oncological treatment is considered a bad prognosis for the disease’s evolution and the patient’s quality of life. In this sense, strategies that can counteract the muscle wasting induced by DOX are essential. In this study, we evaluated the effectiveness of formoterol (FOR), a β2-adrenoceptor agonist, in managing muscle wasting caused by DOX.

**Methods and results:**

To evaluate the effect of FOR on DOX-induced muscle wasting, mice were treated with DOX (2.5 mg/kg b.w., i.p. administration, twice a week), associated or not to FOR treatment (1 mg/kg b.w., s.c. administration, daily). Control mice received vehicle solution. A combination of FOR treatment with DOX protected against the loss of body weight (p<0.05), muscle mass (p<0.001), and grip force (p<0.001) promoted by chemotherapy. FOR also attenuated muscle wasting (p<0.01) in tumor-bearing mice on chemotherapy. The potential mechanism by which FOR prevented further DOX-induced muscle wasting occurred by regulating Akt/FoxO3a signaling and gene expression of atrogenes in skeletal muscle.

**Conclusions:**

Collectively, our results suggest that FOR can be used as a pharmacological strategy for managing muscle wasting induced by DOX. This study provides new insights into the potential therapeutic use of FOR to improve the overall wellbeing of cancer patients undergoing DOX chemotherapy.

## Introduction

A significant challenge in cancer therapy is to develop oncological treatments that promote disease management without negatively affecting the homeostasis of other health systems. Due to its high effectiveness, doxorubicin (DOX) is a chemotherapy drug widely used for cancer treatment (1). However, its use also contributes to muscle wasting (2). Muscle weight loss is a poor prognosis for cancer survival and quality of life (2, 3). Furthermore, patients with increased lean body mass have a better tolerance for chemotherapy treatment (3). Several types of cancers contribute to the development of cachexia (4), which may be potentiated during chemotherapy treatment. DOX is commonly employed to treat several malignancies, including lung cancer (5), breast cancer, lymphoma, and sarcomas (6). Therefore, approaches that promote the maintenance of lean body mass by counter-regulating chemotherapy- and cancer-induced muscle dysfunction are essential.

The molecular mechanism of DOX-induced muscle wasting has been widely discussed in recent decades, with several studies showing intense activation of proteolytic signaling pathways and a decrease in anabolic signaling (7). DOX treatment not only activates the ubiquitin-proteasome system (UPS) (8, 9) and the lysosomal autophagy system (ALS) (10) but also results in a decrease in protein synthesis (11). UPS is a highly regulated process in which E3 ligases play a crucial role in the polyubiquitination of proteins that will be degraded in the proteasome (12). During muscle wasting, there is upregulation of muscle-specific E3 ligases such as muscle ring finger1 (MuRF1/Trim63) and muscle atrophy F-box (Atrogin-1/MAFbx)(13, 14) and more recently demonstrated the role of MUSA1/Fbxo30 and SMART/Fbxo21 (15).

Progress in understanding the mechanisms that lead to the development of muscle atrophy in pathological conditions has enabled advances in interventions to combat muscle wasting. Among them, targeting β2 signaling has been proposed as a potential therapeutic approach to control muscle wasting in different diseases (16, 17). It is known that the activation of β2 receptor signaling promotes muscle mass and function regulation by stimulating pathways that promote muscle protein synthesis and inhibition of protein degradation (17). Formoterol (FOR) is a long-acting β2-adrenoceptor agonist drug, which has been shown to have critical anticachectic effects in experimental models (18). It regulates muscle mass by reducing proteolysis by the ubiquitin-dependent proteolytic system and apoptosis and activating anabolic signaling pathways (18). Furthermore, FOR can decrease muscle wasting in tumor-bearing animals without negatively altering heart function (18, 19). Considering the therapeutic potential of β2-adrenergic agonists for treating muscle wasting, this study aimed to investigate the effectiveness of FOR in reducing muscle wasting caused by DOX chemotherapy and to identify the role of atrogenes in this process.

## Materials and methods

### Animals

Male mice aged between 8 and 10 weeks were used for the *in vivo* experimental protocols. The animals were kept in a room with a light–dark cycle of 12–12 h and a temperature of 22 ± 2°C, with a regular diet and water *ad libitum*. All procedures in this study followed the ethical principles of animal experimentation and were submitted to the Ethics Committee on Animal Experimentation of the University of São Paulo (CEUA No. 4368290920) and the University of Barcelona (CEEA 150/19). All animal manipulations were made in accordance with the European Community guidelines for the use of laboratory animals (20).

### Experimental protocol

Balb/c mice and beta(2)-adrenergic receptor knockout mice (β2-AR −/−) received doxorubicin hydrochloride (DOX) (Eurofarma, Campinas, SP, Brazil) (2.5 mg/kg of b.w., intraperitoneally, twice a week) associated or not to the FOR fumarate dihydrate treatment (Sigma-Aldrich, St. Louis, MO, USA) (1 mg/kg b.w., subcutaneously, daily). Animals that were not submitted to DOX or FOR treatment received saline solution (0.9%) by the corresponding route of administration. The mice were euthanized for sample collection after 4 weeks. Tumor implantation was performed by subcutaneous inoculation of 3.75×10^5^ viable Lewis lung carcinoma (LLC) cells (100 μl) into the right flank of male C57BL/6 mice. Cells were obtained from exponentially growing tumors, and cell viability was determined by Trypan Blue exclusion. The non-tumor-bearing mice group received saline solution (0.9%). These animals then underwent treatment with DOX and FOR, as described above, and after 3 weeks, they were euthanized to obtain tissue samples. Muscles, tumor, and the adipose tissue cushions were weighed and stored at −80°C until analysis.

### Grip‐force assessment

The grip‐force test quantified the strength of the forelimbs. The grip‐force device comprised a pull bar connected to an isometric force transducer (dynamometer). Once the mice were stable holding the bar, they were pulled back into the horizontal plane. Each animal was tested three times, and the average peak of tension was used for the analysis (21).

### C2C12 cell culture

C2C12 cells were maintained in Dulbecco’s modified Eagle’s medium (DMEM, GIBCO, Invitrogen, NY) supplemented with penicillin (100 U/ml), streptomycin (100 µg/ml), and 10% fetal bovine serum (FBS, Atlanta Biological, Lawrenceville, GA). They were grown and maintained in culture bottles at 37°C in a humidified atmosphere containing 5% CO_2_. Upon reaching 90% cell confluence, these cells were subjected to differentiation for 7 days in a 2% horse serum (HS, Biowest, Nuaillé, FR) in DMEM solution. The culture medium was changed every 2 days. Differentiated C2C12 cells received DOX (100 nM) or vehicle solution (DMSO). To evaluate the relevance of β2 adrenergic signaling, part of the cells that received DOX were treated with FOR (200 nM) combined or not with ICI 118,115 hydrochloride (300 nM). The administration of ICI 118,115 (ICI) occurred 30 min before the administration of DOX or FOR. One hour after, the samples were collected for biomolecular analysis. Differentiated C2C12 cells were treated with DOX (100 nM) or vehicle solution (DMSO) to evaluate cell morphology. After 24 h, part of the cells was treated with FOR alone or combined with DOX for another 24 h. Cell morphology was done using Olympus microscopy (Olympus, Inc.). Myotube diameters were measured in at least 200 cells using the ImageJ software (National Institute of Health, Bethesda, MD, USA).

### Gene expression

The samples (gastrocnemius muscle and C2C12 cells) were homogenized in TRIzol reagent (Life Technologies Corporation, Carlsbad, CA, USA) for total RNA extraction following the manufacturer’s recommendations (22). cDNA was synthesized from 2 µg of extracted total RNA using reverse transcriptase (High-Capacity cDNA Reverse Transcription Kit, Thermo Fisher, Carlsbad, CA, USA, 4368814). Gene expression was quantified by real-time PCR (23) using the StepOnePlus Real-Time PCR System (Applied Biosystems, CA, USA) and SYBER Green (Fast SYBR™ Green PCR Master Mix, Applied Biosystems, CA, USA) as a fluorescent label. Gene expression was performed using the comparative Ct method (24), with the expression of Rpl-19 used as an internal control. The sequence of primers used is described in **Supplementary Table S1**.

### Western blotting

The gastrocnemius muscle was homogenized in an extraction buffer containing protease and phosphatase inhibitors (Roche Diagnostics GmbH, Sandhoferstrasse, Mannheim, Germany). After centrifugation, the supernatant was subjected to protein quantification determined by the Bradford assay (Bio-Rad, Hercules, CA, USA) using an albumin standard curve. Samples were diluted in Laemmli’s buffer and submitted to SDS polyacrylamide gel electrophoresis (Sodium dodecyl sulfate (SDS)-PAGE) (25), transferred to a Polyvinylidene fluoride (PVDF) membrane, and incubated with primary antibodies against Akt (ref. 4685), Akt Ser473 (ref. 4058), total FoxO3a (ref. 9467), FoxO3a Ser253 (ref. 9466) (Cell Signaling Technology Danvers, MA, USA), or GAPDH (Santa Cruz Biotechnology, Santa Cruz, CA, USA, ref. SC 25778). They were then incubated with a peroxidase-conjugated anti-IgG antibody and after incubated with the peroxidase substrate (ECL Clarity TM, Bio-Rad, Hercules, CA, USA). GAPDH expression was used as an internal control. Images were obtained using the Amersham Imager 600 equipment (GE Healthcare, Buckinghamshire, UK) and quantified by optical densitometry using Image J software (National Institute of Health, Bethesda, MD, USA).

### Statistical analysis

Statistical analysis was performed using GraphPad Prism software version 6.0 for Windows (GraphPad Software, San Diego, CA, USA). As appropriate, data were analyzed using one- or two-way ANOVA test, followed by the Bonferroni post-test. Data are expressed as mean and standard error. The significance level adopted was p < 0.05.

## Results

### FOR treatment reverses muscle wasting caused by DOX

**Figure 1:**
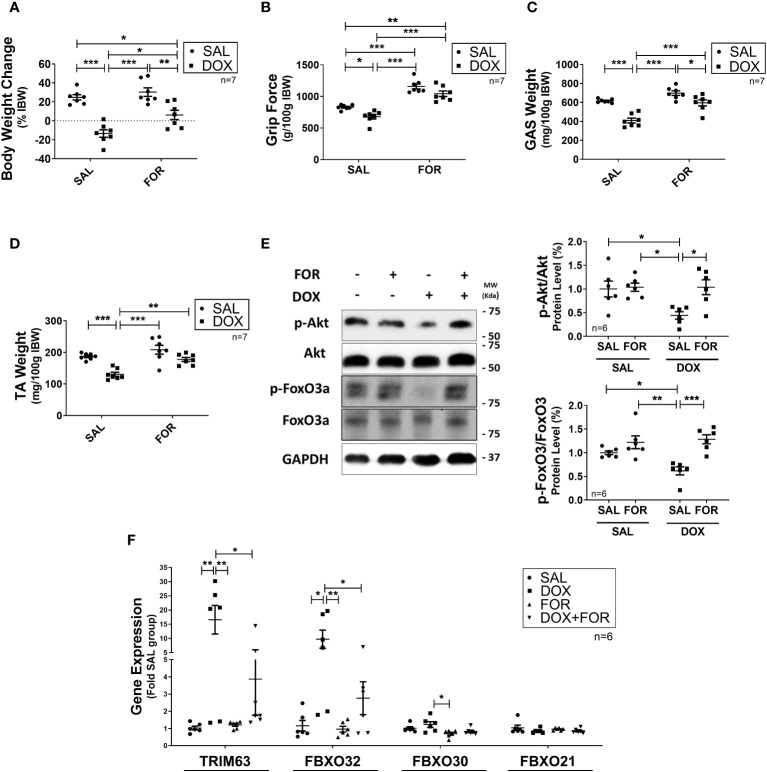
Formoterol inhibits doxorubicin‐induced muscle atrophy. Muscle wasting in Balb/c mice was analyzed by measuring the **(A)** body weight change, **(B)** grip force (g/100g IBW), **(C)** gastrocnemius muscle weight (mg/100g IBW), and **(D)** TA muscle weight (mg/100g IBW). **(E)** FOR prevents the reduction in Akt and FoxO3 phosphorylation caused by DOX, and **(F)** blocks the increase of atrophy‐related gene expression in skeletal muscle. The data are presented as mean ± SEM and analyzed by a two-way ANOVA test followed by the Bonferroni post-test *p < 0.05; **p<0.01; ***p<0.001. DOX, doxorubicin; FOR, formoterol; SAL, saline (0.9%); GAS, gastrocnemius muscle; TA, tibialis anterior muscle; IBW, initial body weight.

We demonstrated that when FOR is added to DOX treatment, it effectively mitigates muscle wasting. Initially, we used a tumor-free *in vivo* experimental model to unravel the isolated effect of chemotherapy on induced atrophy, evaluating underlying molecular pathways and testing a potential therapeutic target. It is well established that DOX treatment activates catabolic pathways in skeletal muscle. We demonstrated that DOX reduced the body weight (p < 0.001) and muscle mass (p < 0.001) of mice submitted to fractionated doses of DOX chemotherapy, also confirmed by the worsening of the functional capacity of the upper limbs (p < 0.05) (**Figures 1A–D**). These effects were, however, abolished by the combination of FOR (**Figures 1A–D**). The DOX treatment decreased the phosphorylation of Akt and FoxO3, an effect that was abolished when the mice received FOR concomitantly (**Figure 1E**). Additionally, FOR treatment protected against DOX-induced increase in Fbxo32 and Trim63 gene expression (**Figure 1F**). The lower food intake promoted by DOX treatment was recovered by FOR treatment (p < 0.001), although it showed no protective effect on the loss of adipose tissue mediated by chemotherapy (p > 0.05) (**Supplementary Table S2**).

### The protection of FOR over DOX-induced muscle catabolism depends on β2 adrenergic activation

**Figure 2:**
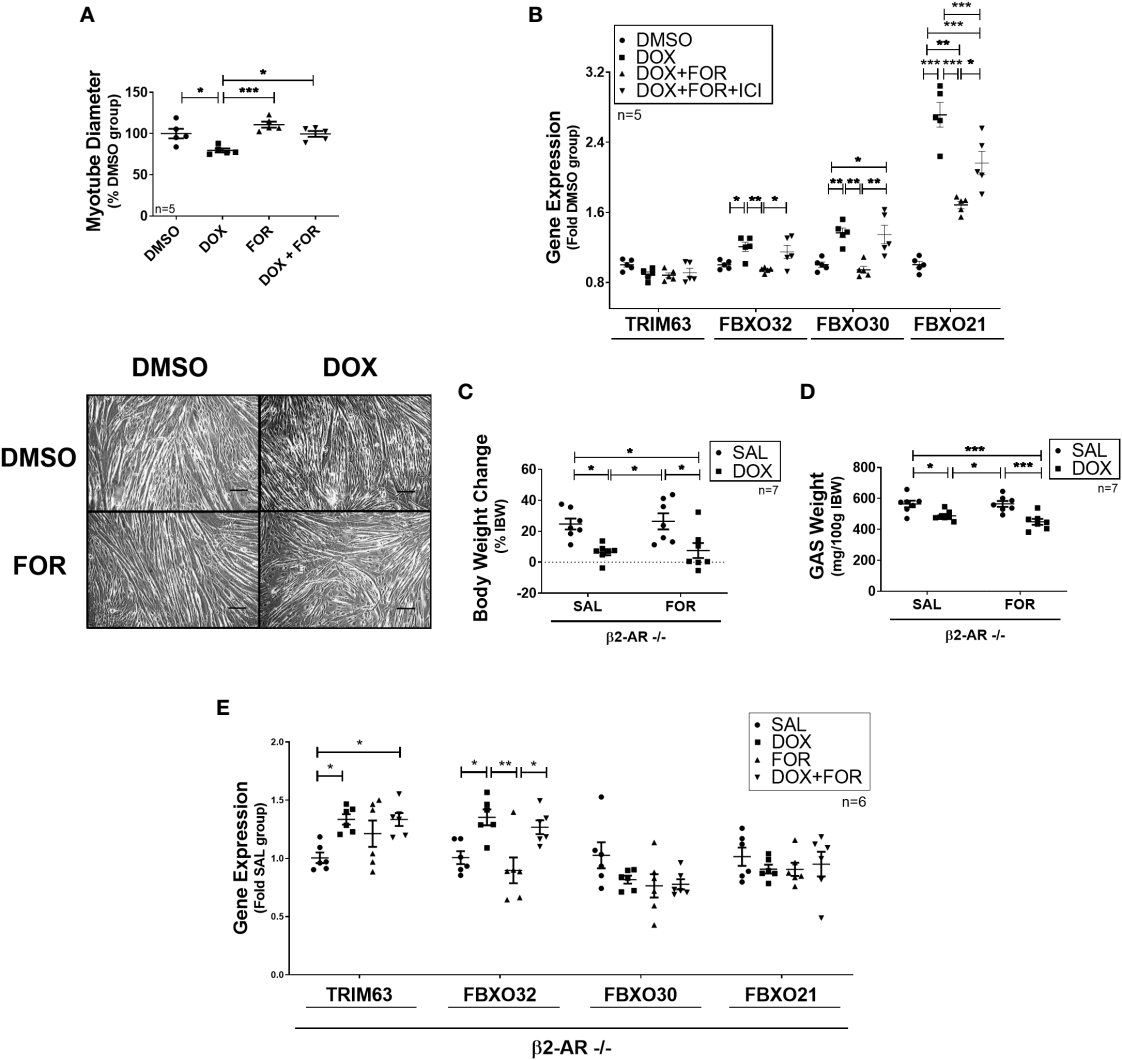
Blockade of β2-adrenergic receptor signaling impairs the atrophy‐related gene downregulation caused by FOR. **(A)** FOR treatment inhibits DOX-induced myotube diameter reduction. Scale bar = 100 μm. **(B)** Downregulation of atrogenes by FOR is partially dependent on β2-adrenergic receptor signaling activation. C2C12 myotubes were submitted to ICI, or vehicle solution, 30 min before DOX and FOR treatment. Muscle wasting in β2-AR −/− mice submitted to DOX and FOR treatment was analyzed by measuring the **(C)** body weight change, **(D)** gastrocnemius muscle weight (mg/100g IBW), and **(E)** atrophy‐related gene expression in skeletal muscle. The data are presented as mean ± SEM and analyzed by one- or two-way ANOVA, followed by the Bonferroni post-test. *p < 0.05; **p<0.01; ***p<0.001. DOX, doxorubicin; FOR, formoterol; SAL, saline (0.9%); GAS, gastrocnemius muscle; IBW, initial body weight; ICI, ICI 118,551.

We then confirmed that FOR treatment prevented the reduction in C2C12 myotube diameter caused by DOX (p<0.05) (**Figure 2A**). In order to better understand this mechanism, we evaluated the effect of the combination of FOR and DOX in the presence of ICI, a potent, selective β2 adrenergic receptor antagonist. While acute treatment with DOX upregulated the gene expression of Fbxo32, Fbxo30, and Fbxo21 (p<0.05), these effects were reversed by the combination with FOR (p <0.05). We also demonstrated that in C2C12 myotubes, the downregulation of Fbxo32 and Fbxo30 gene expression by FOR were entirely dependent on β2 adrenergic activation, while Fbxo21 expression was only partial (**Figure 2B**).

β2-AR −/− animals were then used to confirm the importance of β2 adrenergic activation in FOR protecting from DOX-induced muscle wasting. In these animals, FOR treatment was not able to abolish the body weight and gastrocnemius muscle loss caused by DOX (p > 0.05) (**Figures 2C, D**). In addition, in β2-AR −/− animals, FOR was not able to impair the upregulation of Trim63 and Fbxo32 caused by DOX treatment (**Figure 2E**). Although chemotherapy treatment also caused significant loss of adipose tissue (p<0.001), muscle wasting in β2-AR −/− mice was less sensitive to DOX-induced anorexia and muscle catabolism (**Supplementary Table S3)**.

### FOR also protected against DOX-induced muscle wasting in tumor-bearing mice

**Figure 3:**
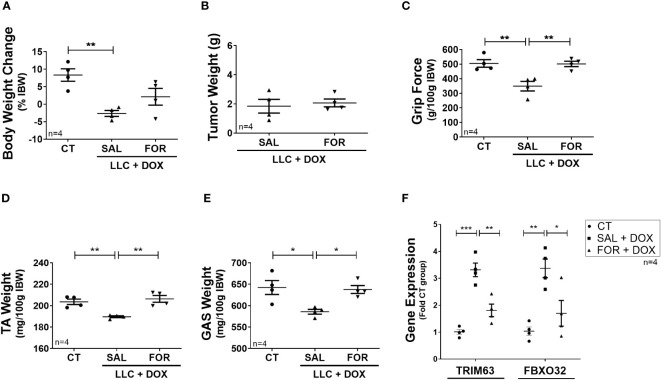
Formoterol inhibits doxorubicin‐induced muscle wasting in tumor-bearing mice. LLC tumor-bearing mice under DOX chemotherapy, associated with or not to FOR treatment, were evaluated by muscle wasting by measuring **(A)** body weight change—without tumor, **(B)** tumor weight, **(C)** grip force (g/100 g IBW), **(D)** TA muscle weight (mg/100g IBW), and **(E)** gastrocnemius muscle weight (mg/100g IBW). **(F)** FOR reduced the DOX-induced Trim63 and Fbxo32 expression. The data are presented as mean ± SEM and analyzed by one-way ANOVA test followed by the Bonferroni post-test. *p < 0.05; **p<0.01; ***p<0.001. DOX, doxorubicin; FOR, formoterol; SAL, saline (0.9%); control group—non-tumor bearing mice (CT); GAS, gastrocnemius muscle; TA, tibialis anterior muscle; IBW, initial body weight.

Finally, we evaluated the efficacy of FOR treatment in an experimental cancer-cachexia model during chemotherapy treatment. The LLC is a widely used experimental cancer model for studying cancer cachexia and evaluating chemotherapeutic agents’ effects. We hypothesized that FOR could also attenuate the muscle mass loss in cachectic mice on chemotherapy. Although FOR did not recover body weight in the presence of DOX treatment (**Figure 3A**) or potentiate the tumor weight reduction (**Figure 3B**), it was able to recover the grip strength and the muscle mass (p<0.05) (**Figure 3C–E**), in addition to reducing the gene expression of Trim63 (p < 0.01) and Fbxo32 (p < 0.05) (**Figure 3F**). As expected, benefits were also observed in the absence of chemotherapy, where the cachexia was minimized by the FOR treatment, which recovered body weight (p <0.05), muscle mass (p < 0.05), and grip force (p < 0.01) caused by the presence of the tumor (**Supplementary Figures S1A–E**), in addition to reducing the gene expression of Trim63 (p < 0.01) and Fbxo32 (p < 0.05) in the skeletal muscle (**Supplementary Figure S1F**).

## Discussion

Over the years, there has been a significant advancement in understanding muscle loss caused by chemotherapy (3, 26–28). Possible therapeutic targets have been studied to alleviate muscle loss during cancer treatment, which extensively impairs the prognosis of the treatment and the quality of life of patients. In the present study, we explored the role of FOR in attenuating muscle wasting caused by DOX chemotherapy. A potential mechanism by which FOR prevents DOX-induced muscle wasting is through the regulation of the Akt/FoxO3a pathway and regulation of expression of atrophy‐related genes in skeletal muscle.

The UPS is a critical system activated during muscle wasting and is responsible for directing proteins to be degraded in the proteasome, including contractile components of skeletal muscle (29). We demonstrated that FOR restores Akt/FoxO3 signaling impaired by DOX treatment, a pathway that has a central role in UPS regulation. DOX chemotherapy impaired Akt signaling, which contributes to reduced FoxO phosphorylation and facilitates FoxO translocation to the nucleus (15, 30). FoxO is a crucial transcription factor that positively regulates the expression of E3 ligases (15), which are responsible for promoting the ubiquitination of proteins directed to the proteasome. MuRF-1/Trim63 and Atrogin1/Fbxo32 are well-known E3 ligases that are upregulated in conditions that lead to muscle wasting, such as those seen in chemotherapy, cancer cachexia, glucocorticoid treatment, and muscle disuse (9, 31).

Prior studies have already shown that DOX chemotherapy reduces insulin sensitivity in skeletal muscle, inhibiting Akt phosphorylation (32) and upregulating the expression of the atrogenes MuRF1 and Atrogin1 (2, 9). However, it is worth noting that some studies reported differences; Nissinen et al. (11) did not detect a significant alteration in MuRF1 expression following DOX treatment (11). The anti-catabolic and hypertrophic effects of FOR are partly mediated by the impingement of β2-adrenergic signaling with insulin/IGF-1 receptor signaling (33). β2-Adrenergic agonists cause increased phosphorylation of Akt, which promotes muscle anabolism through regulation of the Akt/mTOR axis (16, 34). Furthermore, β2-adrenergic receptor signaling can also directly influence skeletal muscle atrophy, mediated by transcriptional modulation of atrogenes under FoxO regulation. Anorexia is a classic clinical symptom observed in cancer cachexia (35). The intervention with FOR also caused a rescue in the food intake of mice treated with DOX, which may have contributed to creating a protective environment, mitigating the adverse effects of DOX on body weight and muscle mass. Here, we demonstrated that FOR protection in DOX-induced upregulation of E3 ligases partly depends on the β2-adrenergic receptor activity. Although a definitive mechanism explaining the differential expression of *in vivo* and *in vitro* approaches remains elusive, a compelling hypothesis revolves around variations in experimental conditions, specifically the chronic treatment *in vivo* versus the acute treatment *in vitro*. In addition to the modulation of the Akt/FoxO3 signaling pathway as a mechanism for protecting muscle wasting in our study, it is crucial to acknowledge that alternative pathways, such as the modulation of protein synthesis and autophagy, may also be implicated in this model.

Previous studies have already demonstrated that β2-adrenergic receptor agonists have an inhibitory effect on the loss of muscle mass in cancer cachexia models (18, 36, 37). By directly affecting skeletal muscle, DOX chemotherapy further complicates the management of cachexia. This is a common condition, as many chemotherapy drugs do not have enough specificity to act only on tumor cells and, consequently, also cause systemic adverse effects (38–41). Our results, however, shed light on the anti-proteolytic effects of FOR during chemotherapy, even during cancer cachexia. Sorafenib chemotherapy treatment, for example, reduces tumor cell content and improves survival in Yoshida AH-130 tumor-bearing rats but does not reduce the cachectic features (42). However, the combination of FOR and megestrol acetate mitigated muscle wasting, improved physical activity, and reduced protein degradation (42). Furthermore, DOX causes a significant loss of white adipose tissue (43), which FOR could not protect. This is due to the importance of the β2-adrenergic receptor signaling for increasing fatty acid mobilization, energy expenditure, and adipogenesis regulation (44, 45). In addition, although we know from experimental data that FOR does not impair cardiac function (19), DOX is known for its cardiotoxic effects (46), so future studies may address these knowledge gaps.

## Conclusions

In conclusion, our findings demonstrate that combining FOR with DOX treatment reduces muscle wasting by mitigating chemotherapy-induced muscle catabolism. Furthermore, FOR treatment exerts its protective effects by regulating Akt/FoxO3a signaling and downregulating the expression of atrogenes in skeletal muscle, although other potential mechanisms may also be involved. These results highlight the significance of FOR treatment as a potential therapeutic strategy to combat muscle wasting during DOX chemotherapy, including cancer cachexia.

## Data availability statement

The raw data supporting the conclusions of this article will be made available by the authors, without undue reservation.

## Ethics statement

The animal study was approved by Ethics Committee on Animal Experimentation of the University of São Paulo (CEUA N° 4368290920) and the University of Barcelona (CEEA 150/19). The study was conducted in accordance with the local legislation and institutional requirements.

## Author contributions

EL, JR and SB: conceptualization. JR, SB, JA, and FL-S: funding acquisition and project administration. EL and JR: methodology. EL and AT: wrote the first draft of the manuscript. EL, AT, LS, QJ, NL, PB, and MP: performed the research and contributed to the interpretation of the results. All authors contributed to the article and approved the submitted version.
