# Supplementary material for: Formoterol reduces muscle wasting in mice undergoing doxorubicin chemotherapy
Source: Front Oncol. 2024 Jan 3;13:1237709. doi: 10.3389/fonc.2023.1237709 (PMC10791811; doi:10.3389/fonc.2023.1237709)
Supplement: Supplementary file 1 [file DataSheet_1.pdf]

Supplementary Material

Formoterol reduces muscle wasting in mice undergoing doxorubicin chemotherapy

Edson Alves de Lima Junior, Alexandre Abilio de Souza Teixeira, Loreana Sanches Silveira; Queralt Jové; Natalia Álvarez Ladrón; Marcelo G. Pereira, Francisco Javier López-Soriano; Josep M Argiles, Patrícia Chakur Brum, Sílvia Busquets Rius, José Cesar Rosa Neto*

*** Correspondence:** Corresponding Author: josecesar23@hotmail.com

Supplementary Table 1. RT-PCR *primer´*s sequence

Gene	Left Primer	Right Primer	GenBank
Trim63	5' GTGTGAGGTGCCTACTTGCTC 3'	5' GCTCAGTCTTCTGTCCTTGGA 3 '	NM_001039048.2
Fbxo32	5' ACAAAGGAAGTACGAAGGAGCG 3'	5' GGCAGTCGAGAAGTCCAGTC 3'	NM_026346.3
Fbxo21	5' GGGCAGGTGAGTCCTCTATG 3'	5' GAGCAGAGACATGCTGATGG 3'	NM_145564.4
Fbxo30	5’ CTTCAGTCTCGTGGAATGGTAA 3’	5’ TGCTCAGGATGTCAGCAAA 3’	NM_027968.3
Rpl19	5' CAATGCCAACTCCCGTCA 3'	5' GTGTTTTTCCGGCAAACGAG 3'	NM_009078.2

Supplementary Table 2. Analysis of muscle mass, adipose tissue weight, and food intake in mice treated with DOX and FOR

Parameters	Experimental groups			
	SAL		FOR	
	SAL (7)	DOX (7)	SAL (7)	DOX (7)
**Food intake (g/day/100g IBW)**	18,31 ± 1,98	15,59 ± 0,53*	22,16 ± 2,96aaa	20,56 ± 2,65aaa
**EDL weight (mg/100g IBW)**	43,05 ± 1,20	26,81 ± 2,73***	44,74 ± 1,97aaa	39,51 ± 1,95aa
**Soleus weight (mg/100g IBW)**	26,24 ± 1,04	17,03 ± 1,52***	29,62 ± 1,72aaa	24,80 ± 1,22aa
**WATe (mg/100g IBW)**	983,91 ± 144,16	313,39 ± 112,81***	606,69 ± 40,73	324,15 ± 92,57***
**WATd (mg/100g IBW)**	265,64 ± 67,02	43,03 ± 19,14**	155,90 ± 15,52	78,92 ± 24,26*

The data are presented as mean ± SEM and analyzed by two-way ANOVA test followed by the Bonferroni post-test. * = $p<0.05$, ** = $p<0.01$, *** = $p<0.001$- vs. control group (SAL); aa = $p<0.01$, aaa = $p<0.001$- vs. DOX group. IBW = initial body weight; WATe = epididymal white adipose tissue; WATd = dorsal white adipose tissue.

Supplementary Table 3. Analysis of muscle mass, adipose tissue weight, and food intake in β2-AR -/- mice treated with DOX and FOR

Parameters	Experimental groups			
	SAL		FOR	
	SAL (7)	DOX (7)	SAL (7)	DOX (7)
**Food intake (g/day/100g IBW)**	19,68 ± 5.03	17,90 ± 1.64	19,60 ± 3,75	19,68 ± 3,80
**Grip Force (g/100g IBW)**	918,34 ± 96,57	771,67 ± 42,26	766,71 ± 36,44	712,19 ± 78,78
**Tibialis Anterior (mg/100g IBW)**	177,83 ± 6,36	163,55 ± 5,42	181,08 ± 6,83	166,36 ± 7,49
**EDL weight (mg/100g IBW)**	35,30 ± 1,25	30,77 ± 1,26	36,24 ± 1,33	30,27 ± 3,46
**Soleus weight (mg/100g IBW)**	24,25 ± 1,02	22,42 ± 1,05	25,11 ± 0,40 [a]	20,64 ± 0,63 [b]
**WATe (mg/100g IBW)**	2167,38 ± 209,20	1074,80 ± 93,15 [***]	2495,70 ± 215,17 [aaa]	862,21 ± 66,03 [***bbb]
**WATd (mg/100g IBW)**	668,33 ± 89,28	263,76 ± 34,47 [***]	881,22 ± 76,25 [aaa]	250,99 ± 39,09 [***bbb]

The data are presented as mean ± SEM and analyzed by two-way ANOVA test followed by the Bonferroni post-test. *** = p<0.001- vs. control group (SAL); a = p<0.05, aaa = p<0.001- vs. DOX group; b = p<0.05, bbb = p<0.001- vs. FOR+SAL group. IBW = initial body weight; WATe = epididymal white adipose tissue; WATd = dorsal white adipose tissue.

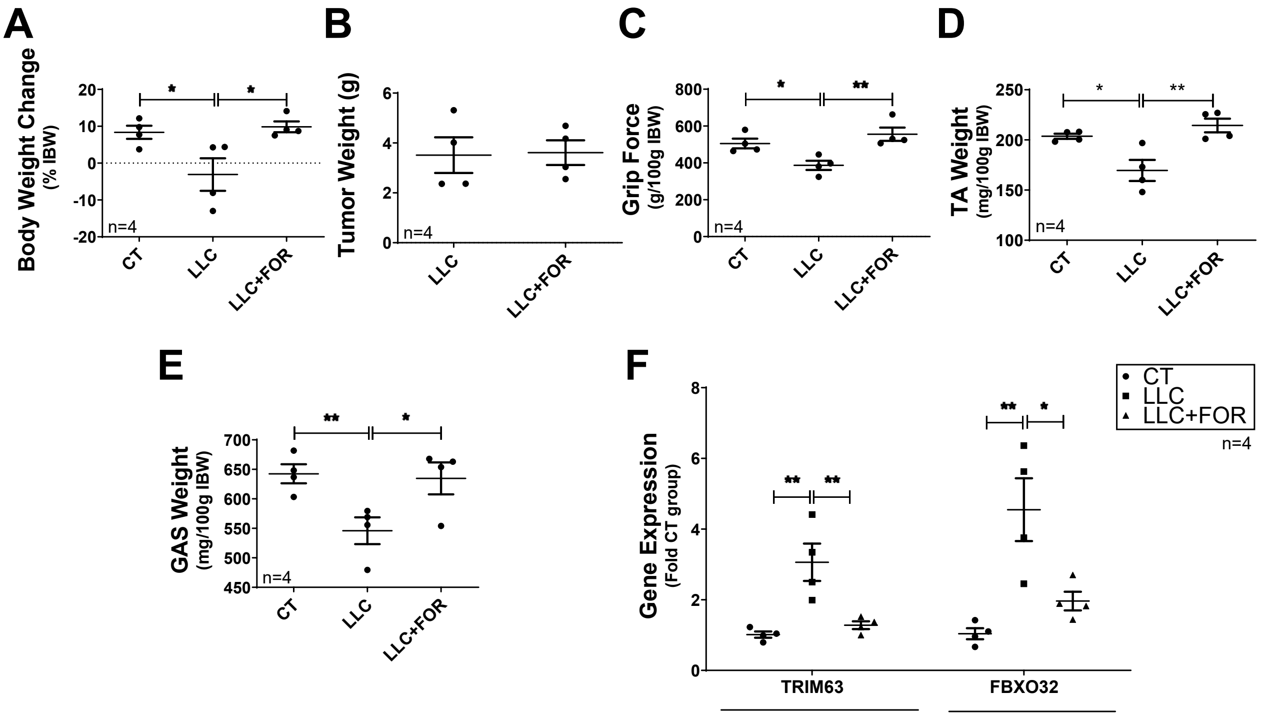

Supplementary Figure 1. Formoterol ameliorates muscle wasting in tumor-bearing mice. Cancer cachexia was analyzed by measuring the (a) body weight change – without tumor, (b) tumor weight, (c) grip force (g/100g IBW), (d) TA muscle weight (mg/100g IBW), (e) Gastrocnemius muscle weight (mg/100g IBW) and (f) atrophy-related genes expression in skeletal muscle. The data are presented as mean ± SEM and analyzed by one-way ANOVA test followed by the Bonferroni post-test. * $p < 0.05$; ** p<0.01; *** p<0.001. DOX, Doxorubicin; FOR, Formoterol; Control group – non-tumor bearing mice (CT); GAS, Gastrocnemius muscle; TA, Tibialis anterior muscle; IBW, Initial body weight.
